# Persistent Inflammation, Immunosuppression, and Catabolism Syndrome and Serious Infections in Burn-Injured Adults: A Retrospective Single-Center Study from 1997 to 2023

**DOI:** 10.1093/jbcr/irag063

**Published:** 2026-04-24

**Authors:** Hannah Kieffer, Michael D Santarelli, Anne L Wagner, Alvin D Jeffery, Ryan J Stark

**Affiliations:** Department of Pediatrics, Vanderbilt University Medical Center, Nashville, TN 37232, United States; Department of Medicine, Vanderbilt University Medical Center, Nashville, TN 37232, United States; Department of Pediatrics, Vanderbilt University Medical Center, Nashville, TN 37232, United States; Department of Surgery, Vanderbilt University Medical Center, Nashville, TN 37232, United States; Department of Biomedical Informatics, Vanderbilt School of Medicine, Nashville, TN 37232, United States; Department of Pediatrics, Vanderbilt University Medical Center, Nashville, TN 37232, United States

**Keywords:** serious bacterial infection, persistent inflammation, immunosuppression, and catabolism syndrome, burn injury

## Abstract

Alterations in both the innate and adaptive immune systems can induce a significant immunocompromised state among those hospitalized after burn injury. These changes can devolve into the clinical entity known as persistent inflammation, immunosuppression, and catabolism syndrome (PIICS), characterized by profound and chronic immune dysregulation. Persistent inflammation, immunosuppression, and catabolism syndrome is defined by the presence of lymphopenia, elevated C-reactive protein levels, and evidence of catabolism through weight loss or hypoalbuminemia in the context of a prolonged hospitalization. Though PIICS has previously been described in patients with burn injuries, this immunophenotype in the setting of serious bacterial and fungal infections is poorly characterized. We performed a detailed retrospective analysis of burn-injured adults (aged 19–64 years) in a de-identified institutional database of patient medical records from 1997 to 2023. Of the 960 patients admitted for a primary burn injury, the overall prevalences of serious infections and PIICS was 38% and 25%, respectively. Both the presence of an inhalation injury and the total body surface area (TBSA) burn size correlated with the development of serious infections and PIICS. Patients with PIICS had more pathogens isolated and a higher prevalence of *Acinetobacter* and *Stenotrophomonas* infections. The immunophenotype of PIICS was strongly associated with recurrent infections during hospitalization. Detailed assessments of PIICS criteria, including trends in lymphocyte counts, may help identify the development of serious bacterial and fungal infections in burn-injured adults.

## INTRODUCTION

In the United States, it is estimated that nearly 30 000 people per year require hospitalization for a burn-related injury.^1^ Extrapolated further, over 8 million cases of burn injury were reported worldwide in 2019.^2^ After survival from the initial burn injury, the risk of inpatient mortality remains in those with moderate to severe injuries, with 50%-60% of deaths attributed to sepsis and subsequent organ dysfunction.^3^^,^^4^ Underlying this risk is the significant impairment in the immune system, both innate and adaptive, instigated by burn injury.^5^ Advances in the acute management of burn injury have improved overall outcomes but, despite these advances, mortality rates have recently remained stagnant around 3%.^6^^,^^7^ In some instances, this immune dysfunction leads to a protracted hospital course with refractory immunological aberrancies and impaired recovery known as persistent inflammation, immunosuppression, and catabolism syndrome (PIICS).^7^^,^^8^

First described in surgical patients, the immunophenotype of PIICS has since been expanded to a more general, critically ill patient population with diverse underlying pathophysiology.^9^^,^^10^ Persistent inflammation, immunosuppression, and catabolism syndrome is characterized by certain laboratory criteria in patients who have had an intensive care unit (ICU) stay of at least 10-14 days or a hospital stay greater than 14 days; it requires a C-reactive protein (CRP) level of greater than 5 or 15 mg/dL, an absolute lymphocyte count (ALC) of less than 800 cells/μL, and a weight loss greater than 10% or albumin levels less than 3 g/dL.^8^^,^^9^ There remains a degree of heterogeneity in the diagnostic criteria and laboratory cutoffs used in different studies but the key components remain consistent with those outlined above.^9^^,^^11^ Persistent inflammation, immunosuppression, and catabolism syndrome generates a persistent and dysfunctional state of enhanced innate immunity, suppressed adaptive immunity, and increased energy needs that propagate prolonged hospitalization, often resulting in late mortality.^12^ While globally described in critically ill patients, different underlying disease states have unique, population-specific risk factors that contribute to this state of persistent immune dysfunction. Patients with burn injuries have consistently been shown to remain in a prolonged hypermetabolic state with an increased risk of serious infection. However, the burden of PIICS and its risk factors have been poorly characterized in this population.

To this end, we aimed to determine the overall prevalence of PIICS in burn-injured adults with one or more culture-positive infections and the associated immune cell profiles. Through this, we aimed to gain a deeper understanding of the immunological trajectory of these vulnerable patients using standard values measured by treating providers to aid in the understanding of the necessary surveillance and consequences of PIICS, including subsequent infections.

## MATERIALS AND METHODS

### Participants

We conducted a retrospective, descriptive cohort study at a single academic medical center using electronic health record data. We collected data from the Vanderbilt University Medical Center (VUMC) Synthetic Derivative (SD), a de-identified version of the electronic health record spanning over 25 years (between 1997 and 2023).^13^ Approval was granted by the VUMC Institutional Review Board (#192203). The selection criteria focused on records within the system with an associated International Classification of Diseases (ICD) 9 or 10 code, or a Current Procedural Terminology (CPT) code related to burn injuries or care (Index e1). A manual review of individual records followed the initial data extraction to confirm that patients were between 19 and 64 years old at the time of admission. Age was restricted to less than 65 due to the known enhanced mortality in older burned individuals and to lessen the impact of age-related immune dysfunction on PIICS estimates.^14^^,^^15^ Additional manual extraction from narrative data captured admission and discharge dates, percent total body surface area (TBSA) burned, the mechanism of injury, the presence of inhalation injury, concomitant trauma, comorbidities, and mortality during the hospitalization. Mortality was considered related to the burn injury if it occurred during the initial inpatient stay; deaths after discharge from the primary burn-related hospitalization were not included. If a patient had more than one hospitalization for burn injury, only the initial burn hospitalization was included. Patients admitted for the treatment of desquamating skin disorders, such as Stevens–Johnson syndrome or toxic epidermal necrolysis, were excluded (Figure e1).

### Serious bacterial/fungal infection determination and culture data

Serious bacterial or fungal infections, termed serious infections for this manuscript, are defined as infections requiring hospital admission with an increased risk of complications, such as sepsis and subsequent organ dysfunction.^16-18^ The presence of serious bacterial or fungal infections was determined based on ICD codes (Index e2) and culture positivity. Patients in our serious bacterial or fungal infection cohort must have met at least one of the following conditions: a positive blood culture, a positive respiratory culture while on mechanical ventilation, or a documented diagnosis of sepsis in their chart with a known pathogen.^19^^,^^20^ Though wound and urine cultures were noted, they were not necessarily linked to a serious infectious event unless stated in the chart or associated with another positive culture that was temporally and species-related at a different culture site. The dates of positive cultures, the sources of the culture data, and the types of organisms present were manually extracted and recorded.

### PIICS determination and white blood cell counts

Patients were classified as having PIICS if they fulfilled 4 or more of the specified laboratory and clinical criteria: a hospital stay exceeding 14 days, a CRP level above 10 mg/L, an ALC of fewer than 1000 cells/μL, and signs of somatic protein catabolism indicated by either more than 10% weight loss at any time during hospitalization based on admission weight or hypoalbuminemia, defined as an albumin level below 3.0 g/dL or a prealbumin level below 10 g/dL. A lymphocyte cutoff of < 1000 cells/μL was used based on the National Institutes of Health definition of lymphopenia. Patients meeting 3 of these criteria, where one value was missing due to a lack of collected data, were also included in the PIICS population. In addition, white blood cell differential counts were obtained for each individual patient throughout their hospitalization.

### Statistical analysis

We used percentages to describe categorical data and medians with interquartile ranges (IQRs) to describe continuous data. Nonparametric categorical data were assessed using chi-square or Fisher’s exact tests. Nonparametric continuous data were analyzed through a Mann–Whitney *U*-test. We used multivariable logistic regression analyses to evaluate how age, sex, TBSA burn size, inhalational injury, and burn mechanism correlated with both the development of serious infections and PIICS. Univariate logistic regression analyses examined the relationship between lymphopenia, CRP, somatic protein catabolism, and the development of serious infections. To inspect lab value trends over time, centered, third-order (cubic), nonlinear regression lines with 95% likelihood confidence intervals for the respective median laboratory values were utilized for ALC, CRP, and albumin, with curve comparisons conducted using the extra-sum-of-squares F test. All presented and analyzed data followed the STROBE guidelines. Data analysis was executed using Python 3.12 (Python Software Foundation, Fredericksburg, VA) and GraphPad Prism 10 (GraphPad Software Inc., La Jolla, CA). Results were deemed statistically significant at a *P*-value of < .05.

## RESULTS

### Patient characteristics

Of the 1895 patients identified within the initial SD chart extraction, 960 patients between the ages of 19 and 64 were identified as having a primary admission related to a burn injury (Figure e1). Patients with burn injuries who had a serious infectious event accounted for 38% of the total number of admissions, and patients who developed PIICS accounted for 25%. Though the number of patients who were burn-injured was predominantly male (78% vs 22%, *P* = .04), there were no differences in sex, age, or race/ethnicity between patients who had either a serious infection or PIICS compared to those who did not. A greater proportion of those with a serious infection or PIICS had an inhalational injury or larger percent burn TBSA compared to those without (Table 1).

**Table 1:** Characteristics of Patients With or Without PIICS or Serious Infection

	**Total** **(*n* = 960)**	**PIICS** **(*n* = 235)**	**No PIICS** **(*n* = 725)**	***P* value**	**Serious infection (*n* = 367)**	**No serious infection** **(*n* = 593)**	***P* value**
**Gender**				.097			.033
Male	752 (78%)	175 (74%)	577 (80%)		274 (75%)	478 (81%)	
Female	208 (22%)	60 (26%)	148 (20%)		93 (25%)	115 (19%)	
**Age** (years)	36 (28-44)	37 (28-43)	35 (27-45)	.987	37 (29-43)	35 (27-45)	.759
**Hospital length of stay** (days)	15 (7-33)	39 (29-62)	10 (5-21)	<.001	36 (20-57)	9 (4-17)	<.001
**Race**				.734			.27
White	783 (82%)	189 (80%)	594 (82%)		290 (79%)	493 (83%)	
Black	95 (10%)	23 (10%)	72 (10%)		40 (11%)	55 (9%)	
Other	82 (8%)	23 (10%)	59 (18%)		37 (10%)	45 (8%)	
**Ethnicity**				.489			.332
Hispanic/Latino	37 (4%)	12 (5%)	25 (3%)		18 (5%)	19 (3%)	
Non-Hispanic/Latino	923 (96%)	223 (95%)	700 (97%)		349 (95%)	574 (97%)	
**Presence of inhalational injury**	276 (29%)	99 (42%)	177 (24%)	<.001	163 (44%)	113 (19%)	<.001
**Mechanism of burn**				.306			<.001
Flame	814 (85%)	201 (86%)	613 (85%)		319 (87%)	495 (83%)	
Scald	58 (6%)	10 (4%)	48 (7%)		12 (3%)	46 (8%)	
Electrical	39 (4%)	13 (6%)	26 (3%)		22 (6%)	17 (3%)	
Other	49 (5%)	11 (4%)	38 (5%)		14 (4%)	35 (6%)	
**TBSA size**				<.001			<.001
<20%	218 (23%)	22 (9%)	196 (27%)		37 (10%)	181 (31%)	
20%-49%	583 (60%)	148 (63%)	435 (60%)		226 (62%)	357 (60%)	
>50%	159 (17%)	65 (28%)	94 (13%)		104 (28%)	55 (9%)	
**Comorbidities**							
Neurologic	75 (7%)	23 (10%)	52 (7%)	.194	32 (9%)	43 (7%)	.421
Respiratory	65 (7%)	5 (2%)	60 (8%)	.001	21 (6%)	44 (7%)	.301
Cardiovascular	128 (13%)	27 (11%)	101 (14%)	.338	41 (11%)	87 (15%)	.115
Gastrointestinal	34 (4%)	6 (3%)	28 (4%)	.345	13 (4%)	21 (4%)	.990
Endocrine	57 (6%)	15 (6%)	42 (6%)	.739	27 (7%)	30 (5%)	.148
Nephrology	5 (1%)	0 (0%)	5 (1%)	.202	1 (0%)	4 (1%)	.398
Hematologic or oncologic	4 (0%)	0 (0%)	4 (1%)	.253	0 (0%)	4 (1%)	.114
Obstetric or gynecologic	7 (1%)	2 (1%)	5 (1%)	.800	3 (1%)	4 (1%)	.805
Rheumatologic	10 (1%)	0 (0%)	10 (1%)	<.001	1 (0%)	9 (2%)	<.001
MSK or trauma	62 (6%)	16 (7%)	46 (6%)	.802	21 (6%)	41 (7%)	.455
Psychiatric	147 (15%)	37 (16%)	110 (15%)	.832	57 (16%)	90 (15%)	.905
Substance abuse	316 (33%)	68 (29%)	248 (34%)	.135	110 (30%)	206 (35%)	.116
Obesity	28 (3%)	11 (5%)	17 (2%)	.257	17 (5%)	11 (2%)	.257

Abbreviations: MSK, musculoskeletal; PIICS, persistent inflammation, immunosuppression, and catabolism syndrome.

Data are presented as number (%) or median (interquartile range).

Substance abuse includes the use of alcohol, tobacco, and illegal substances.

### Sources, types of pathogens, and timing of serious infectious events

**Figure 1:**
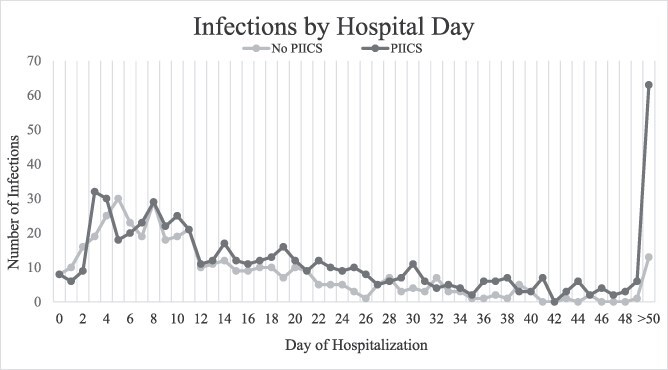
Total Number of Infections Between Patients With (Black) and Without PIICS (Grey) by Hospital Day*.* Abbreviations: PIICS = persistent inflammation, immunosuppression, and catabolism syndrome

Among patients with documented positive culture results, 358 (81%) patients met our criteria for a serious infection. Across all patients, the median time to culture positivity was 6 days (Figure 1). Of the patients who had a culture-positive event during their hospitalization, 257 (58%) had one within the first week. We found that patients who went on to develop PIICS had shorter times to initial infection than those who did not, but the difference did not reach statistical significance (median time 6 days vs 7 days, *P* = .07). When examining clinical isolates, 1708 individual pathogen results were identified (Table 2). The raw number of isolates was significantly higher in patients who developed PIICS across all identified organisms, with a mean of 2.7 infections per patient in the PIICS group compared to 0.6 in the non-PIICS group (*P* < .001). When examining specific species or organism classes, patients who developed PIICS were more likely to have a Gram-negative or fungal species compared to a Gram-positive species (OR 1.3; 95% CI, 1.05-1.6; *P* = .017). At the individual-species level, patients who developed PIICS had more isolates of *Bacillus* species, *Pseudomonas* species, *Acinetobacter baumannii*, and *Stenotrophomonas maltophilia* than patients who did not develop PIICS (Table 2).

**Table 2:** Top 30 Isolates by Number

**Organism isolated**	**PIICS isolates** **(*n* = 1016)**	**No PIICS isolates** **(*n* = 692)**	**Total isolates** **(*n* = 1708)**	**OR**	**95% CI**	***P* value**
**Gram-positive organisms**						
* Staphylococcus aureus*	151	121	272	0.82	0.63-1.07	.16
* Streptococcus pneumoniae*	17	21	38	0.54	0.28-1.04	.07
* Enterococcus faecalis*	16	9	25	1.21	0.53-2.76	.79
* Enterococcus* NOS	18	9	27	1.37	0.61-3.07	.69
* Clostridium difficile*	10	2	12	3.45	0.75-15.71	.55
* Staphylococcus* coagulase negative	9	11	20	0.55	0.23-1.34	.25
* Streptococcus pyogenes*	9	7	16	0.87	0.32-2.36	.80
* Bacillus* species NOS	7	0	7	10.29	0.59-180.6	.05
* Staphylococcus epidermidis*	5	3	8	1.14	0.27-4.77	1.00
Gram-positive cocci NOS	6	13	19	0.31	0.12-0.82	.02
Total Gram-positive isolates	270	221	491	0.77	0.62-0.95	.03
**Gram-negative organisms**						
* Acinetobacter baumannii*	113	46	159	1.76	1.22-2.51	.002
* Pseudomonas aeruginosa*	109	70	179	1.07	0.78-1.47	.75
* Enterobacter cloacae*	53	41	94	0.87	0.57-1.33	.59
* Stenotrophomonas maltophilia*	47	7	54	4.75	2.13-10.6	<.001
* Escherichia coli*	45	35	80	0.91	0.58-1.43	.73
* Klebsiella pneumoniae*	33	26	59	0.86	0.51-1.46	.59
* Haemophilus influenzae*	30	27	57	0.75	0.44-1.27	.34
* Acinetobacter* NOS	24	10	34	1.65	0.78-3.47	.22
* Serratia marcescens*	22	17	39	0.88	0.46-1.67	.74
* Enterobacter aerogenes*	19	10	29	1.30	0.60-2.81	.57
* Enterobacter* NOS	18	17	35	0.72	0.37-1.40	.38
* Pseudomonas* NOS	18	36	54	0.33	0.19-0.58	.001
* Proteus mirabilis*	11	4	15	1.90	0.60-5.60	.30
* Klebsiella oxytoca*	6	8	14	0.51	0.18-1.47	.27
* Klebsiella* NOS	6	7	13	0.58	0.19-1.74	.40
Gram-negative rods NOS	6	9	15	0.45	0.16-1.28	.19
Total Gram-negative isolates	631	407	1038	1.15	0.94-1.40	.17
**Fungal species**						
* Candida albicans*	54	30	84	1.24	0.78-1.96	.43
* Candida parapsilosis*	13	7	20	1.27	0.50-3.20	.66
* Fusarium* species	12	4	16	2.06	0.66-6.40	.31
Yeast	7	6	13	0.79	0.27-2.37	.78
* Candida* NOS	5	7	12	0.48	0.15-1.53	.24
Total fungal isolates	115	64	179	1.25	0.91-1.73	.20

Abbreviations: NOS, not otherwise specified; OR, odds ratio; PIICS, persistent inflammation, immunosuppression, and catabolism syndrome

### Trends in PIICS laboratory criteria and cell count differentials over time

**Figure 2:**
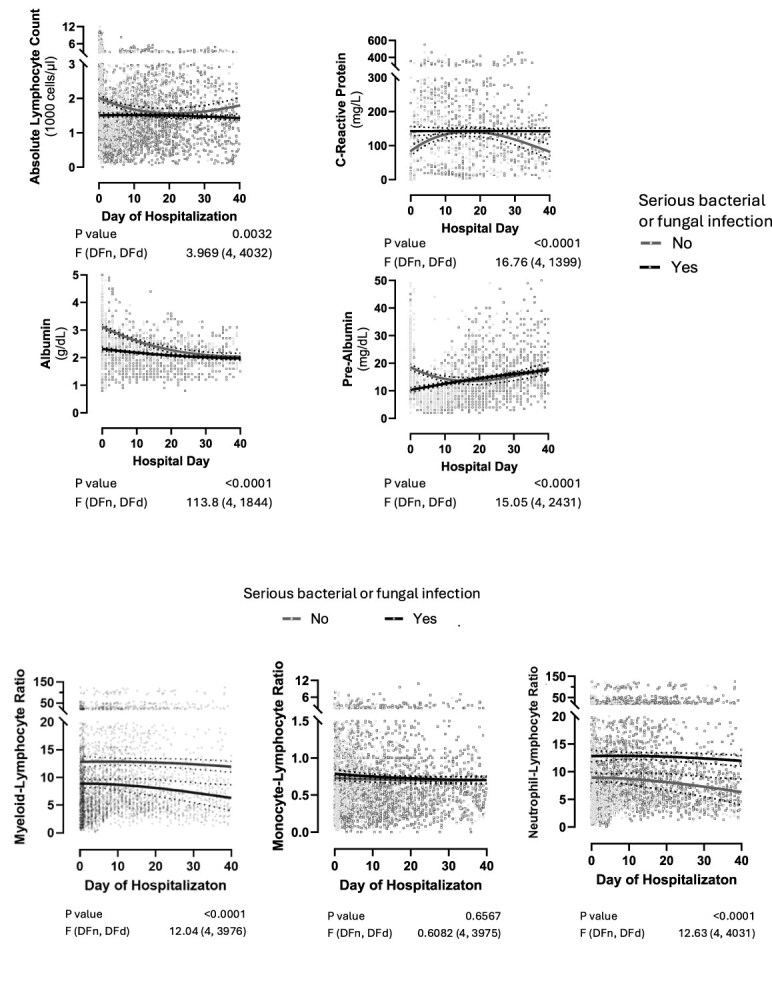
Daily Lab Trends With Nonlinear, Third-Order Regression Lines of Respective Median Lab Value (With 95 % CI Likelihood) for Patients Postburn With Serious Bacterial or Fungal Infection (Black, Squares) and Patients Postburn Without Serious Bacterial or Fungal Infection (Grey, Circles)

A detailed examination of PIICS-related laboratory trends was performed for patients with and without a serious infection (Figure 2). Patients with a serious infection had lower initial ALC (*P* = .015), albumin (*P* < .001), and prealbumin (*P* = .001) than patients without. The admission CRP was not significantly different between patients who developed and did not develop a serious infection (*P* = .551). For patients who did not, ALC declined and CRP rose, reaching a nadir or peak, respectively, between weeks 2 and 3 of hospitalization, before recovering. Alternatively, those who suffered from serious infections had persistently lower ALCs and higher CRPs that did not significantly change by day 40 of hospitalization. For both albumin and prealbumin, while patients who developed serious infections had lower overall values on admission, both groups reached similar numbers by day 40 with a faster convergence in prealbumin. When examining white blood cell ratios, neutrophil-to-lymphocyte ratios (NLRs) were more elevated at presentation and remained elevated throughout hospitalization in those with serious infections (*P* < .001), whereas monocyte-to-lymphocyte ratios remained globally unchanged between groups (*P* = .66). The addition of all myeloid lineage cells (basophils, eosinophils) compared to lymphocyte counts showed a similar trend and significance as that of NLR alone. In addition, a higher NLR on admission corresponded to an increased number of infections during admission (median NLR by number of infections; 0 infections = 6.33, 1-2 infections = 6.96, ≥ 3 infections = 9.62, *P* = .006).

### Association of clinical and laboratory variables with serious infections or PIICS

To determine which clinical and laboratory variables and, specifically, which laboratory components of PIICS were most associated with serious infections at any time during hospitalization, a univariate logistic regression analysis was performed. The presence of an inhalational injury and TBSA burn size had the strongest associations with a serious infectious event (Table 3). While other characteristics, such as age, sex, and most mechanisms of injury, were not associated with the development of serious infections, scald injuries were found to be associated with reduced rates when referenced against flame injuries (scald injury: OR 0.4, *P* = .006). Patients who went on to meet the clinical and laboratory criteria of PIICS were also more likely to experience a serious infectious event (OR 12; 95% CI, 8.51-17.54; *P* < .001). When examining the associations between the individual PIICS criteria and serious infections, all criteria were found to be significantly associated. Evidence of somatic catabolism was found to have the strongest association (Table 3; evidence of somatic catabolism: OR 7.97; 95% CI, 5.57-11.67; *P* < .001), followed by lymphopenia.

**Table 3:** Separate Multivariable Logistic Regression Analysis of Patient Characteristics and PIICS Criteria With the Development of Serious Bacterial or Fungal Infections

**Variable**	**Odds ratio**	**95% Confidence interval**	***P* value**
Gender	0.71	0.52-0.97	.033
Age (years)	0.99	0.99-1.01	.765
Percent TBSA (%)	1.04	1.03-1.04	<.001
Presence of inhalational injury	3.42	2.56-4.59	<.001
**Burn mechanism (Flame as a reference)**			
Scald	0.40	0.20-0.75	.006
Other	1.07	0.68-1.67	.771
**PIICS criteria**			
Lymphopenia^a^	5.03	3.79-6.72	<.001
Evidence of somatic catabolism^b^	7.97	5.57-11.67	<.001
CRP	2.73	1.06-7.91	.046

Abbreviations: CRP, C-reactive protein; LOS, length of stay; PIICS, persistent inflammation, immunosuppression, and catabolism syndrome; TBSA, total body surface area.

a Evidence of lymphopenia defined as white blood cell count < 1000 cells/μL.

b Evidence of somatic protein catabolism defined as presence of either albumin < 3.0 g/dL, or prealbumin < 10 g/dL or weight loss > 10% from admission weight at any point during admission.

## DISCUSSION

The inflammatory response to burn injury involves both pro- and anti-inflammatory reactions, engaging both the innate and adaptive immune systems.^21^^,^^22^ The main treatment strategies to reduce this response include early burn wound surgical excision and grafting to promote wound closure.^23^^,^^24^ Providing nutritional support to meet the energy needs of the immune system and facilitate wound healing is also important for improving outcomes.^25^ Ideally, these interventions limit the damage and the persistence of injury to the tissue, thereby mitigating the extent of immune system activation after a burn and enabling a more rapid return to immunological homeostasis. However, when immune balance cannot be restored, a prolonged state of immune dysfunction called PIICS can develop.^8^ This dysregulated state is characterized by an imbalance among immune cells, such as neutrophils (due to increased granulopoiesis), at the expense of adaptive immunity. This immunologic impairment has been shown to increase the risk of adverse responses to secondary exposures, including severe bacterial and fungal infections, which can lead to sepsis accompanied by significant morbidity and mortality.^13^^,^^26^^,^^27^

In this study, we examined the prevalence of PIICS and severe bacterial and fungal infections in adult patients with burn injuries aged 19-64 admitted over the past 25 years to a large, tertiary burn referral center. Within this cohort, 25% of the patients developed a PIICS phenotype and 38% experienced a serious infectious event, including 187 patients (19% of the entire cohort) with both a serious infection and PIICS. Patients who went on to develop PIICS had more pathogens isolated, specifically a higher number of Gram-negative and fungal species, than those who did not develop PIICS. Patients with PIICS had a higher NLR on admission that persisted throughout their hospitalization, and this elevated admission NLR correlated with a greater number of unique pathogenic episodes. In the regression analysis, the presence of inhalational injury, larger burn TBSA, and the development of PIICS were independently associated with serious infections. This is consistent with previously published data on PIICS in other hospitalized patients.^28^ The data demonstrate that the laboratory and metabolic derangements that occur in the setting of PIICS and serious infections are more significant than those caused by burn injuries alone. This highlights the utility of laboratory data in clarifying the complex clinical picture and identifying which patients are at an increased risk of these complications.

The mechanism behind the development of PIICS is multifaceted, and burn injury introduces unique contributing pathways. Burn injury triggers a sterile, noninfectious inflammatory response driven by damage-associated molecular patterns released from injured tissue, activating innate antigen-presenting cells and cytokine networks.^29^ This process parallels immune dysregulation following trauma, with early pro-inflammatory signaling followed by counter-regulation.^30^ However, burn injuries specifically, as demonstrated in this article and other research, are often complicated by infection because of the immune system dysregulation and the breakdown of the skin barrier.^31^ As such, the immune system can also be further activated by pathogen-associated molecular patterns.^32^ This activation is best characterized by systemic inflammatory response syndrome (SIRS). A compensatory anti-inflammatory response syndrome (CARS) is triggered simultaneously and serves to counteract SIRS. The two states, when equally unyielding, can coexist leading to PIICS.^33^^,^^34^

Persistent inflammation, immunosuppression, and catabolism syndrome describes a chronic critical illness phenotype characterized by ongoing inflammation and immune suppression with profound catabolism.^8^^,^^12^ This manifests as sustained hypermetabolism with insulin resistance and accelerated protein breakdown, contributing to muscle and organ injury.^35^^,^^36^ The proposed mechanistic pathway above suggests that injury and infection trigger the development of PIICS. Our data and prior work done in this field, however, suggest that it is not this simple. There is evidence that patients who develop PIICS are more likely to present with markers consistent with an immunosuppressed and catabolic state on admission, with the opposite phenotype being protective against PIICS.^37^^,^^38^ This parallels the research done on low weight and infection risk, demonstrating that severe infections stimulate a hypermetabolic state that increases a patient’s rate of catabolism and weight loss, but also that a low body mass index increases one’s risk of developing an infection.^39^ Furthermore, our research shows the presence of PIICS increases a patient’s risk of recurrent infections, demonstrating that the relationship is bidirectional. It is likely that certain patient characteristics, present at the time of the injury or infectious trigger, predispose those patients to PIICS and increase their risk of its eventual development.

While there are no standardized treatments for PIICS, interventions that target each contributing component and attempt to break the deadlock between SIRS and CARS have been suggested.^11^ Medications aimed at re-establishing immune homeostasis are being considered, such as granulocyte-macrophage colony-stimulating factor, which restores monocytes and has been studied in the setting of sepsis, and granulocyte colony-stimulating factor, which may restore lymphocyte counts but has never been evaluated in critically ill populations.^40^^,^^41^ Interleukin (IL)-1 receptor blockade with anakinra or IL-6 antibodies has been shown in certain patient populations to reduce inflammatory signaling.^42^ Gemcitabine, a pyrimidine analog, has also been suggested to modulate myeloid-derived suppressor cell (MDSC) activation and prevent deleterious, persistent inflammation after the acute injuries and infections in animal models.^43^ However, careful timing and selection of broad immunosuppression are critical given the significantly elevated risk of infections in patients with burn injuries, especially those with PIICS. In addition, targeting the catabolic processes associated with PIICS through physical therapy and nutritional support has been shown to be beneficial.^23^ Medical management to address catabolism has been explored, including the use of IGF-1/IGFBP-3, which has been shown to reduce muscle breakdown in patients with burn injuries.^44^ More research is needed to determine whether these treatments could provide clinical benefit to patients with PIICS.

This study has several limitations that should be acknowledged. First, the cohort of patients identified was based on data extraction linked to ICD-9 and ICD-10 codes, which can misidentify patients and miss patients who met the criteria but were not coded as such. Second, because data were recorded at the discretion of the treating team and then collected retrospectively, there were missing values for related outcomes of interest, potentially leading to underestimations of the prevalence or effect size for PIICS. Furthermore, based on the retrospective nature of this paper and the limitations of our dataset, we were unfortunately unable to determine the prevalence of sepsis in our cohort owing to its definition based on clinical criteria not available to extract from our dataset and not laboratory or culture data. As a surrogate, we chose to look at PIICS in the setting of serious bacterial and fungal infections, which are associated with an increased risk of sepsis and organ dysfunction. Third, because laboratory trends are dynamic within cohorts and individual patients, we cannot determine specific laboratory cutoff values to predict infections or PIICS development. Fourth, we chose to use a lymphocyte cutoff of < 1000 cells/μL, rather than the previously published 800 cells/μL. This was chosen based on the National Institutes of Health definition of lymphopenia, but this may have led to overestimations of PIICS in older individuals.^45^ Furthermore, due to the nature of the database used, we were not able to reliably determine the ICU length of stay (LOS) for each patient. As a surrogate, we used hospital length of stay alone. While there are definitions that allow either ICU LOS or hospital LOS, we acknowledge that ICU LOS is more often used in the literature.^9^ Fifth, because the data were collected over a span of 2 decades, changes in practice or treatments may have affected the outcomes, and these could not be adjusted for. Lastly, as this was a single-center retrospective study, there may be unintentional practice bias or institutional confounders that could limit the generalizability of the assessments and outcomes to other centers.

## CONCLUSION

In analyzing a database of adult patients with burn injuries admitted to a single institution over the past 25 years, we found an overall prevalence of PIICS of approximately 1 in 4 patients. As expected, both inhalation injury and burn TBSA were strongly linked to the development of serious bacterial and fungal infections and PIICS. Patients who developed PIICS experienced more infections compared to those without, specifically with an increased number of Gram-negative and fungal infections. The laboratory markers of PIICS such as lymphopenia, elevated CRP, and evidence of catabolism through low albumin/prealbumin levels were all demonstrated to be present early in the hospitalization and were significantly associated with earlier and more frequent infections. This suggests that the early presence of related biomarkers could aid in early risk screening for both serious infections and PIICS in this patient population. Further research into these laboratory markers, including lymphocyte ratios, is necessary to better understand what the predisposing factors are for the development of sepsis and PIICS are. This may allow for earlier identification and targeted treatment.

## Supplementary Material

Supplemental_Materials_irag063

## Notes

Author contributions: Hannah Kieffer (Data curation, Formal analysis, Investigation [equal], Methodology [supporting], Writing—original draft, Writing—review & editing [lead]), Michael Santarelli (Data curation, Formal analysis, Writing—review & editing [equal]), Anne Wagner (Conceptualization, Methodology [supporting], Writing—review & editing [equal]), Alvin Jeffery (Conceptualization [supporting], Data curation, Formal analysis [equal], Methodology [supporting], Writing—review & editing [equal]), and Ryan Stark (Conceptualization, Data curation [equal], Formal analysis [supporting], Investigation, Methodology, Software [equal], Supervision [lead], Writing—original draft, Writing—review & editing [equal]). H. Kieffer and M.D. Santarelli contributed equally.

Funding: We gratefully acknowledge the support from these funding sources. Dr Hannah Kieffer is a member of the Vanderbilt MPRINT Precision Therapeutics Academy, which is supported by the Eunice Kennedy Shriver National Institute of Child Health and Human Development of the National Institutes of Health under award number P50HD106446. Dr Alvin Jeffery is supported by the Gordon and Betty Moore Foundation through grant GBMF9048. Dr Ryan Stark is supported by the National Institute of General Medical Sciences of the National Institutes of Health under award number R35GM138191. The content of this manuscript is solely the responsibility of the authors and does not necessarily represent the official views of the National Institutes of Health or the Gordon and Betty Moore Foundation.
