# Supplementary material for: Persistent Inflammation, Immunosuppression, and Catabolism Syndrome and Serious Infections in Burn-Injured Adults: A Retrospective Single-Center Study from 1997 to 2023
Source: J Burn Care Res. 2026 Apr 24;47(4):1195–203. doi: 10.1093/jbcr/irag063 (PMC13338637; doi:10.1093/jbcr/irag063)
Supplement: Supplemental_Materials_irag063 [file supplemental_materials_irag063.docx]

Persistent Inflammation, Immunosuppression, and Catabolism Syndrome and Sepsis in Burn-Injured Adults: A Retrospective Single Center Study from 1997-2023

Hannah Kieffer, Michael D. Santarelli, Anne L. Wagner, Alvin D. Jeffery, Ryan J. Stark

Supplement Materials

Figure:

- Figure e1: Breakdown of inclusion and exclusion criteria in determination of burn-injured adults with PIICS

Index:

- Index e1: Burn Specific Billing Codes
- Index e2: Sepsis Specific Billing Codes


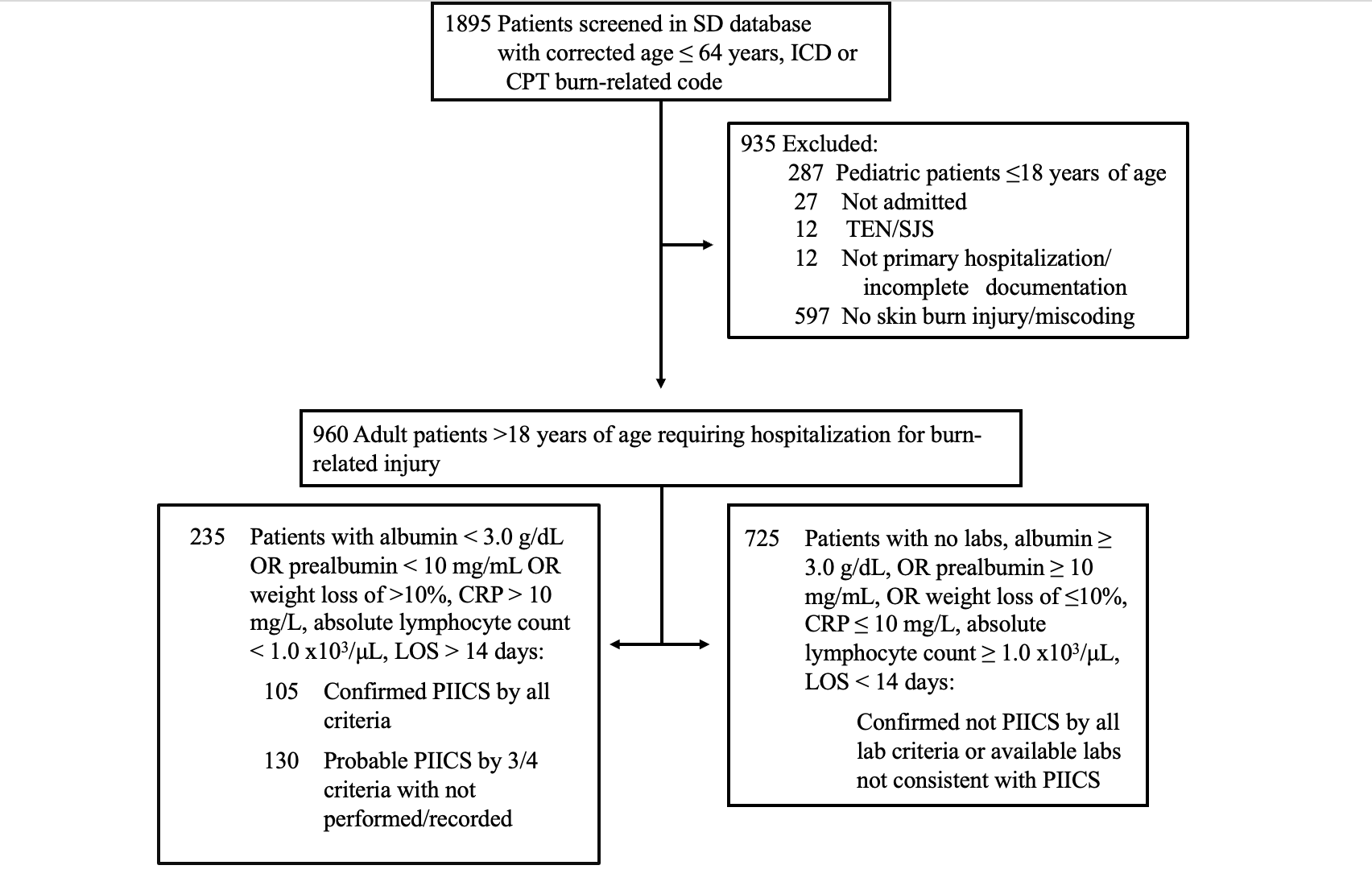
**Figure e1: Flowchart of inclusion and exclusion criteria and sub-phenotype breakdown**

**Index e1: Burn Specific Billing Codes**

ICD9 code 941.3-Full-thickness skin loss due to burn [third degree NOS] of face, head, and neck

ICD9 code 942.3-Full-thickness skin loss due to burn [third degree NOS] of trunk

ICD9 code 943.3-Full-thickness skin loss due to burn [third degree NOS] of upper limb, except wrist and hand

ICD9 code 944.3-Full-thickness skin loss due to burn [third degree NOS] of wrist(s) and hand(s)

ICD9 code 945.3-Full-thickness skin loss due to burn [third degree NOS] of lower limb(s)

ICD9 code 948.20-Burn [any degree] involving 20-29 percent of body surface with third degree burn, less than 10 percent or unspecified

ICD9 code 948.21-Burn [any degree] involving 20-29 percent of body surface with third degree burn, 10-19%

ICD9 code 948.22-Burn [any degree] involving 20-29 percent of body surface with third degree burn, 20-29%

ICD9 code 948.2-Burn [any degree] involving 20-29 percent of body surface

ICD9 code 948.30-Burn [any degree] involving 30-39 percent of body surface with third degree burn, less than 10 percent or unspecified

ICD9 code 948.31-Burn [any degree] involving 30-39 percent of body surface with third degree burn, 10-19%

ICD9 code 948.32-Burn [any degree] involving 30-39 percent of body surface with third degree burn, 20-29%

ICD9 code 948.33-Burn [any degree] involving 30-39 percent of body surface with third degree burn, 30-39%

ICD9 code 948.3-Burn [any degree] involving 30-39 percent of body surface

ICD9 code 948.40-Burn [any degree] involving 40-49 percent of body surface with third degree burn, less than 10 percent or unspecified

ICD9 code 948.41-Burn [any degree] involving 40-49 percent of body surface with third degree burn, 10-19%

ICD9 code 948.42-Burn [any degree] involving 40-49 percent of body surface with third degree burn, 20-29%

ICD9 code 948.43-Burn [any degree] involving 40-49 percent of body surface with third degree burn, 30-39%

ICD9 code 948.44-Burn [any degree] involving 40-49 percent of body surface with third degree burn, 40-49%

ICD9 code 948.4-Burn [any degree] involving 40-49 percent of body surface

ICD9 code 948.50-Burn [any degree] involving 50-59 percent of body surface with third degree burn, less than 10 percent or unspecified

ICD9 code 948.51-Burn [any degree] involving 50-59 percent of body surface with third degree burn, 10-19%

ICD9 code 948.52-Burn [any degree] involving 50-59 percent of body surface with third degree burn, 20-29%

ICD9 code 948.53-Burn [any degree] involving 50-59 percent of body surface with third degree burn, 30-39%

ICD9 code 948.54-Burn [any degree] involving 50-59 percent of body surface with third degree burn, 40-49%

ICD9 code 948.55-Burn [any degree] involving 50-59 percent of body surface with third degree burn, 50-59%

ICD9 code 948.5-Burn [any degree] involving 50-59 percent of body surface

ICD9 code 948.60-Burn [any degree] involving 60-69 percent of body surface with third degree burn, less than 10 percent or unspecified

ICD9 code 948.61-Burn [any degree] involving 60-69 percent of body surface with third degree burn, 10-19%

ICD9 code 948.62-Burn [any degree] involving 60-69 percent of body surface with third degree burn, 20-29%

ICD9 code 948.63-Burn [any degree] involving 60-69 percent of body surface with third degree burn, 30-39%

ICD9 code 948.64-Burn [any degree] involving 60-69 percent of body surface with third degree burn, 40-49%

ICD9 code 948.65-Burn (any degree) involving 60-69 percent of body surface with third degree burn, 50-59%

ICD9 code 948.66-Burn [any degree] involving 60-69 percent of body surface with third degree burn, 60-69%

ICD9 code 948.6-Burn [any degree] involving 60-69 percent of body surface

ICD9 code 948.70-Burn [any degree] involving 70-79 percent of body surface with third degree burn, less than 10 percent or unspecified

ICD9 code 948.71-Burn [any degree] involving 70-79 percent of body surface with third degree burn, 10-19%

ICD9 code 948.72-Burn [any degree] involving 70-79 percent of body surface with third degree burn, 20-29%

ICD9 code 948.73-Burn [any degree] involving 70-79 percent of body surface with third degree burn, 30-39%

ICD9 code 948.74-Burn [any degree] involving 70-79 percent of body surface with third degree burn, 40-49%

ICD9 code 948.75-Burn [any degree] involving 70-79 percent of body surface with third degree burn, 50-59%

ICD9 code 948.76-Burn [any degree] involving 70-79 percent of body surface with third degree burn, 60-69%

ICD9 code 948.77-Burn [any degree] involving 70-79 percent of body surface with third degree burn, 70-79%

ICD9 code 948.7-Burn [any degree] involving 70-79 percent of body surface

ICD9 code 948.80-Burn [any degree] involving 80-89 percent of body surface with third degree burn, less than 10 percent or unspecified

ICD9 code 948.81-Burn [any degree] involving 80-89 percent of body surface with third degree burn, 10-19%

ICD9 code 948.82-Burn [any degree] involving 80-89 percent of body surface with third degree burn, 20-29%

ICD9 code 948.83-Burn [any degree] involving 80-89 percent of body surface with third degree burn, 30-39%

ICD9 code 948.84-Burn [any degree] involving 80-89 percent of body surface with third degree burn, 40-49%

ICD9 code 948.86-Burn [any degree] involving 80-89 percent of body surface with third degree burn, 60-69%

ICD9 code 948.85-Burn [any degree] involving 80-89 percent of body surface with third degree burn, 50-59%

ICD9 code 948.87-Burn [any degree] involving 80-89 percent of body surface with third degree burn, 70-79%

ICD9 code 948.88-Burn [any degree] involving 80-89 percent of body surface with third degree burn, 80-89%

ICD9 code 948.8-Burn [any degree] involving 80-89 percent of body surface

ICD9 code 948.90-Burn [any degree] involving 90 percent or more of body surface with third degree burn, less than 10 percent or unspecified

ICD9 code 948.91-Burn [any degree] involving 90 percent or more of body surface with third degree burn, 10-19%

ICD9 code 948.92-Burn [any degree] involving 90 percent or more of body surface with third degree burn, 20-29%

ICD9 code 948.93-Burn [any degree] involving 90 percent or more of body surface with third degree burn, 30-39%

ICD9 code 948.94-Burn [any degree] involving 90 percent or more of body surface with third degree burn, 40-49%

ICD9 code 948.95-Burn [any degree] involving 90 percent or more of body surface with third degree burn, 50-59%

ICD9 code 948.96-Burn [any degree] involving 90 percent or more of body surface with third degree burn, 60-69%

ICD9 code 948.97-Burn [any degree] involving 90 percent or more of body surface with third degree burn, 70-79%

ICD9 code 948.98-Burn [any degree] involving 90 percent or more of body surface with third degree burn, 80-89%

ICD9 code 948.99-Burn [any degree] involving 90 percent or more of body surface with third degree burn, 90% or more of body surface

ICD9 code 948.9-Burn [any degree] involving 90 percent or more of body surface

ICD10 code T27.0XXA-Burn of larynx and trachea, initial encounter

ICD10 code T27.0XXD-Burn of larynx and trachea, subsequent encounter

ICD10 code T27.0XXS-Burn of larynx and trachea, sequela

ICD10 code T27.1XXA-Burn involving larynx and trachea with lung, initial encounter

ICD10 code T27.2XXA-Burn of other parts of respiratory tract, initial encounter

ICD10 code T27.1XXD-Burn involving larynx and trachea with lung, subsequent encounter

ICD10 code T27.1XXS-Burn involving larynx and trachea with lung, sequela

ICD10 code T27.2XXD-Burn of other parts of respiratory tract, subsequent encounter

ICD10 code T27.2XXS-Burn of other parts of respiratory tract, sequela

ICD10 code T27.3XXA-Burn of respiratory tract, part unspecified, initial encounter

ICD10 code T27.3XXA-Burn of respiratory tract, part unspecified, initial encounter

ICD10 code T27.3XXD-Burn of respiratory tract, part unspecified, subsequent encounter

ICD10 code T27.3XXS-Burn of respiratory tract, part unspecified, sequela

ICD10 code T31.20-Burns involving 20-29% of body surface with 0% to 9% third degree burns

ICD10 code T31.21-Burns involving 20-29% of body surface with 10-19% third degree burns

ICD10 code T31.22-Burns involving 20-29% of body surface with 20-29% third degree burns

ICD10 code T31.30-Burns involving 30-39% of body surface with 0% to 9% third degree burns

ICD10 code T31.31-Burns involving 30-39% of body surface with 10-19% third degree burns

ICD10 code T31.32-Burns involving 30-39% of body surface with 20-29% third degree burns

ICD10 code T31.33-Burns involving 30-39% of body surface with 30-39% third degree burns

ICD10 code T31.40-Burns involving 40-49% of body surface with 0% to 9% third degree burns

ICD10 code T31.41-Burns involving 40-49% of body surface with 10-19% third degree burns

ICD10 code T31.42-Burns involving 40-49% of body surface with 20-29% third degree burns

ICD10 code T31.43-Burns involving 40-49% of body surface with 30-39% third degree burns

ICD10 code T31.50-Burns involving 50-59% of body surface with 0% to 9% third degree burns

ICD10 code T31.44-Burns involving 40-49% of body surface with 40-49% third degree burns

ICD10 code T31.51-Burns involving 50-59% of body surface with 10-19% third degree burns

ICD10 code T31.52-Burns involving 50-59% of body surface with 20-29% third degree burns

ICD10 code T31.53-Burns involving 50-59% of body surface with 30-39% third degree burns

ICD10 code T31.54-Burns involving 50-59% of body surface with 40-49% third degree burns

ICD10 code T31.55-Burns involving 50-59% of body surface with 50-59% third degree burns

ICD10 code T31.60-Burns involving 60-69% of body surface with 0% to 9% third degree burns

ICD10 code T31.61-Burns involving 60-69% of body surface with 10-19% third degree burns

ICD10 code T31.62-Burns involving 60-69% of body surface with 20-29% third degree burns

ICD10 code T31.63-Burns involving 60-69% of body surface with 30-39% third degree burns

ICD10 code T31.64-Burns involving 60-69% of body surface with 40-49% third degree burns

ICD10 code T31.65-Burns involving 60-69% of body surface with 50-59% third degree burns

ICD10 code T31.66-Burns involving 60-69% of body surface with 60-69% third degree burns

ICD10 code T31.70-Burns involving 70-79% of body surface with 0% to 9% third degree burns

ICD10 code T31.72-Burns involving 70-79% of body surface with 20-29% third degree burns

ICD10 code T31.73-Burns involving 70-79% of body surface with 30-39% third degree burns

ICD10 code T31.74-Burns involving 70-79% of body surface with 40-49% third degree burns

ICD10 code T31.75-Burns involving 70-79% of body surface with 50-59% third degree burns

ICD10 code T31.71-Burns involving 70-79% of body surface with 10-19% third degree burns

ICD10 code T31.76-Burns involving 70-79% of body surface with 60-69% third degree burns

ICD10 code T31.77-Burns involving 70-79% of body surface with 70-79% third degree burns

ICD10 code T31.80-Burns involving 80-89% of body surface with 0% to 9% third degree burns

ICD10 code T31.81-Burns involving 80-89% of body surface with 10-19% third degree burns

ICD10 code T31.82-Burns involving 80-89% of body surface with 20-29% third degree burns

ICD10 code T31.83-Burns involving 80-89% of body surface with 30-39% third degree burns

ICD10 code T31.84-Burns involving 80-89% of body surface with 40-49% third degree burns

ICD10 code T31.85-Burns involving 80-89% of body surface with 50-59% third degree burns

ICD10 code T31.86-Burns involving 80-89% of body surface with 60-69% third degree burns

ICD10 code T31.87-Burns involving 80-89% of body surface with 70-79% third degree burns

ICD10 code T31.88-Burns involving 80-89% of body surface with 80-89% third degree burns

ICD10 code T31.90-Burns involving 90% or more of body surface with 0% to 9% third degree burns

ICD10 code T31.91-Burns involving 90% or more of body surface with 10-19% third degree burns

ICD10 code T31.92-Burns involving 90% or more of body surface with 20-29% third degree burns

ICD10 code T31.93-Burns involving 90% or more of body surface with 30-39% third degree burns

ICD10 code T31.94-Burns involving 90% or more of body surface with 40-49% third degree burns

ICD10 code T31.95-Burns involving 90% or more of body surface with 50-59% third degree burns

ICD10 code T31.96-Burns involving 90% or more of body surface with 60-69% third degree burns

ICD10 code T31.97-Burns involving 90% or more of body surface with 70-79% third degree burns

ICD10 code T31.98-Burns involving 90% or more of body surface with 80-89% third degree burns

ICD10 code T31.99-Burns involving 90% or more of body surface with 90% or more third degree burns

CPT code 15852

**Index e2: Sepsis Specific Billing Codes:**

ICD10 code A22.7-Anthrax sepsis

ICD10 code A26.7-Erysipelothrix sepsis

ICD10 code A32.7-Listerial sepsis

ICD10 code A40.0-Sepsis due to streptococcus, group A

ICD10 code A40.1-Sepsis due to streptococcus, group B

ICD10 code A40.3-Sepsis due to Streptococcus pneumoniae

ICD10 code A40.8-Other streptococcal sepsis

ICD10 code A40.9-Streptococcal sepsis, unspecified

ICD10 code A41.01-Sepsis due to Methicillin susceptible Staphylococcus aureus

ICD10 code A41.02-Sepsis due to Methicillin resistant Staphylococcus aureus

ICD10 code T81.44XA-Sepsis following a procedure, initial encounter

ICD10 code A41.2-Sepsis due to unspecified staphylococcus

ICD10 code A41.3-Sepsis due to Hemophilus influenzae

ICD10 code A41.1-Sepsis due to other specified staphylococcus

ICD10 code A41.50-Gram-negative sepsis, unspecified

ICD10 code A41.51-Sepsis due to Escherichia coli [E. coli]

ICD10 code A41.52-Sepsis due to Pseudomonas

ICD10 code A41.53-Sepsis due to Serratia

ICD10 code A41.59-Other Gram-negative sepsis

ICD10 code A41.81-Sepsis due to Enterococcus

ICD10 code A41.89-Other specified sepsis

ICD10 code A41.9-Sepsis, unspecified organism

ICD10 code A42.7-Actinomycotic sepsis

ICD10 code A54.86-Gonococcal sepsis

ICD10 code B37.7-Candidal sepsis

ICD10 code P36.8-Other bacterial sepsis of newborn

ICD10 code P36.9-Bacterial sepsis of newborn, unspecified

ICD10 code R65.20-Severe sepsis without septic shock

ICD10 code R65.21-Severe sepsis with septic shock

ICD9 code 670.2-Puerperal sepsis

PheWAS code group 994-Sepsis and SIRS

PheWAS code group 994.2-Sepsis

ICD10 code A02.1-Salmonella sepsis

ICD9 code 995.92-Severe sepsis

ICD9 code 995.91-Sepsis

ICD10 code A41.4-Sepsis due to anaerobes
